# The Cutting Edge of Affinity Electrophoresis Technology

**DOI:** 10.3390/proteomes3010042

**Published:** 2015-03-18

**Authors:** Eiji Kinoshita, Emiko Kinoshita-Kikuta, Tohru Koike

**Affiliations:** Department of Functional Molecular Science, Institute of Biomedical and Health Sciences, Hiroshima University, Kasumi 1-2-3, Hiroshima 734-8553, Japan; E-Mails: kikuta@hiroshima-u.ac.jp (E.K.-K.); tkoike@hiroshima-u.ac.jp (T.K.)

**Keywords:** affinity electrophoresis, capillary affinity electrophoresis, affinity-trap polyacrylamide gel electrophoresis, saccharide affinity electrophoresis, supported molecular matrix electrophoresis, phosphate affinity electrophoresis, phos-tag, posttranslational modification

## Abstract

Affinity electrophoresis is an important technique that is widely used to separate and analyze biomolecules in the fields of biology and medicine. Both quantitative and qualitative information can be gained through affinity electrophoresis. Affinity electrophoresis can be applied through a variety of strategies, such as mobility shift electrophoresis, charge shift electrophoresis or capillary affinity electrophoresis. These strategies are based on changes in the electrophoretic patterns of biological macromolecules that result from interactions or complex-formation processes that induce changes in the size or total charge of the molecules. Nucleic acid fragments can be characterized through their affinity to other molecules, for example transcriptional factor proteins. Hydrophobic membrane proteins can be identified by means of a shift in the mobility induced by a charged detergent. The various strategies have also been used in the estimation of association/disassociation constants. Some of these strategies have similarities to affinity chromatography, in that they use a probe or ligand immobilized on a supported matrix for electrophoresis. Such methods have recently contributed to profiling of major posttranslational modifications of proteins, such as glycosylation or phosphorylation. Here, we describe advances in analytical techniques involving affinity electrophoresis that have appeared during the last five years.

## 1. Introduction

Affinity electrophoresis is a separation technique that is based on the phenomenon whereby the mobilities of charged molecules in a direct-current electric field vary according to whether or not they interact with other molecules for which they have affinities. Affinity electrophoresis has become an important technique, especially in the biological and medical sectors, where it is used to separate and analyze biological macromolecules. The first affinity electrophoresis technique, cross electrophoresis, was developed by Nakamura *et al.* [1,2]. Cross electrophoresis has been used in the detection of enzyme-substrate complexes [3,4,5,6] and in analyses of the kinetics of antigen-antibody reactions [7,8]. The principle associated with this method has been applied in the techniques of rocket immunoelectrophoresis and crossed immunoelectrophoresis [9].

A wide variety of affinity electrophoresis techniques have been developed that provide good qualitative results or have good separation capabilities. One such pioneering technique is that of affinophoresis [10], which utilizes an affinophore, a charged hydrophilic polymer that binds to molecules with a suitable affinity. In affinophoresis, only molecules that bind specifically to the affinophore can be induced to migrate by application of an electric field. The development of affinity probes (or affinity ligands), which are molecules that have affinities for specific molecules, now occupies an important role in the growth of affinity electrophoresis technology. In addition, affinity electrophoresis techniques based on the retardation of the migration of molecules that bind strongly to affinity probes immobilized on a supported matrix have also been developed, and these have contributed to analyses of major posttranslational modifications of proteins [11,12,13,14]. Various types of apparatus for affinity electrophoresis have been developed for use in such techniques as capillary electrophoresis [15,16,17] and microchip electrophoresis [18,19,20]. Capillary affinity electrophoresis, in particular, has been widely used as a technique for various modes in the field of life science [21]. Moreover, affinity electrophoresis is also used in a new electrophoresis technique in which a poly (vinylidene fluoride) (PVDF) membrane is used as a novel supported molecular matrix [22,23,24]. Attempts have been made to apply this technique in affinity separations of large glycoproteins [25].

This article describes advances in analytical techniques involving affinity electrophoresis that have appeared during the last five years. In addition, it introduces a technique for the analysis of phosphorylated proteins that is based on the phosphate-affinity probe, Phos-tag [11,26,27,28,29,30,31,32,33], which we developed, and it describes the contributions made to phosphoproteomics by techniques based on Phos-tag.

## 2. Capillary Affinity Electrophoresis

**Figure 1:**
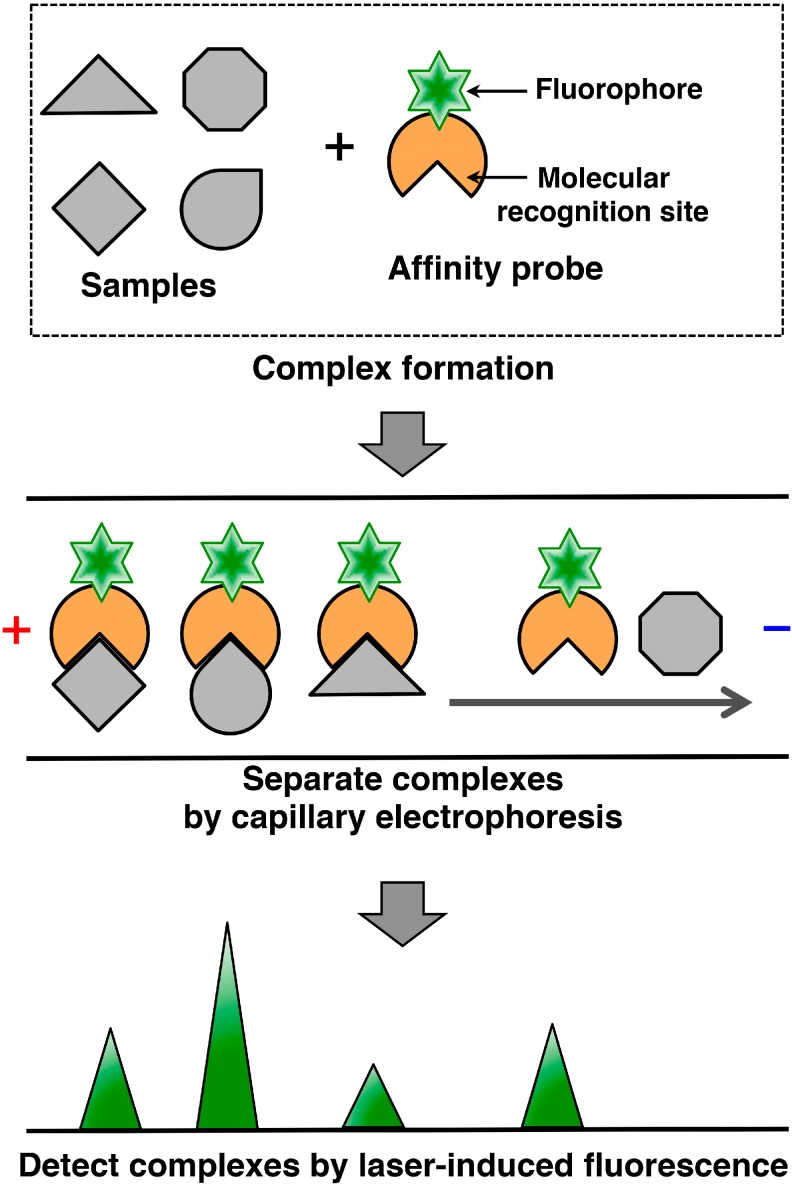
Schematic representation of capillary affinity electrophoresis (CAE). Fluorescently-labeled affinity probes are mixed with the sample, and the resultant complexes are separated by capillary electrophoresis. The principle of separation is the change in mobility resulting from the difference in the dynamic equilibrium between the target molecules and the affinity probes in the electrophoretic field. This method also permits the electrical migration of only those molecules that are strongly bound to the affinity probes.

Capillary affinity electrophoresis (CAE) is a method that combines the use of intermolecular interactions in free solutions with capillary electrophoresis. In CAE, fluorophore-labeled molecules that have affinities for the target molecules are used as affinity probes. The affinity probes and samples are mixed, and the resulting complexes are separated by capillary electrophoresis. The method operates on the principle that the mobility of the target molecules changes as a result of intermolecular interactions (Figure 1). A detection system based on scanning laser-induced fluorescence permits detection with high sensitivity and high precision, even with minute amounts (<1 μL) of sample. This section is focused on advances in CAE techniques involving fluorescent labeling of affinity molecules.

Shimura and Karger used a fluorophore-labeled fragment antigen-binding (Fab) region of immunoglobulin purified from a monoclonal antibody as an affinity probe to perform CAE of a human growth hormone [15]. Because the uniformity of electric charges is important in enhancing the specificity of the labeled Fab to a target molecule, Shimura and Karger fluorescently labeled a specific cysteine residue on the Fab region and then purified it before use. By this method, deamidated species of human growth hormone were successfully separated. Shimura and Kasai improved the preparation of the affinity probe by the use of an expensive low-yield monoclonal antibody, and they described a new preparation method that uses a recombinant antibody [16]. The new method with the recombinant antibody permits the introduction of cysteine residues for fluorescent labeling, the substitution of amino acid residues to control electric charges and large-scale preparations. Shimura and Kasai also prepared an affinity probe from a recombinant antibody against human insulin, and they performed CAE of insulin. Although the complex of the affinity probe and insulin dissociated rapidly with a half-life of only a few minutes, the complex was successfully detected because the electrophoresis was completed within five minutes.

Phillips and Wellner developed a microchip-based capillary electrophoresis system for the separation of antigens in a sample [17]. They immobilized antibodies onto a glass filter and inserted the filter into the injection port of a microchip. Antigens in the sample were captured and fluorescently labelled in the glass filter and subsequently eluted and separated by capillary electrophoresis. Six chemokines associated with neuroinflammation in premature infants were successfully separated and quantified from cerebrospinal fluid samples within ten minutes. The fact that the concentrations of these chemokines were in the region of 10 pg/mL proved that this method is highly sensitive.

One advantage of CAE is that affinity probes can be prepared for various molecules ranging from macromolecules (such as antibodies) to low-molecular-weight compounds. Another advantage is that CAE separations can be completed with short migration times, permitting the detection of short-lived complexes that cannot be detected by other methods. Consequently, CAE should have a range of further applications in analyses of various intermolecular interactions.

## 3. Affinity-Trap Polyacrylamide Gel Electrophoresis

**Figure 2:**
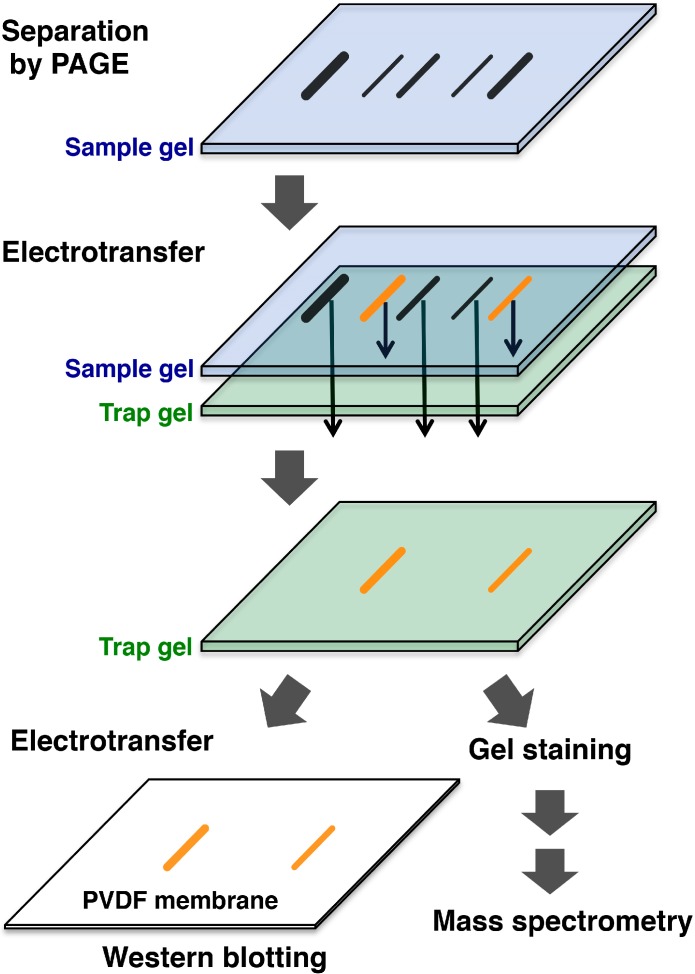
Schematic representation of affinity-trap polyacrylamide gel electrophoresis (AT-PAGE). Protein samples obtained from biological specimens are separated by normal PAGE and then transferred to a trapping gel on which affinity probes are immobilized. Proteins that do not have an affinity for the affinity probes pass through the trapping gel. Proteins that interact specifically with the affinity probes and are trapped by the trapping gel are immediately detected and can be easily identified by means of Western blotting or mass spectrometry.

Polyacrylamide gel electrophoresis (PAGE) has become the most-widely adopted method for the separation of proteins, because it has good separation abilities and because it can be used in combination with various other analytical methods, such as Western blotting, amino acid sequence analysis or mass spectrometry, once the proteins have been transferred to the PVDF membrane. Awada *et al.* developed affinity-trap PAGE (AT-PAGE), a technique that exploits the properties of a polyacrylamide gel [34]. In this method, protein samples obtained from biological specimens are separated by PAGE and then transferred to a polyacrylamide gel (the affinity-trap gel) on which affinity probes are immobilized. In the transfer step, proteins that do not have an affinity for the affinity probes pass through the affinity-trap gel. Proteins trapped by the affinity-trap gel can be visualized by gel staining and identified by mass spectrometry after in-gel digestion (Figure 2). By using trypsin as an affinity probe and a crude extract of soybean flour as a sample, Awada *et al.* succeeded in isolating and identifying a trypsin inhibitor [34]. AT-PAGE is likely to be useful in analyses of differences in the expression of specific proteins and in analyses of posttranslational modifications of proteins, as well as in the isolation and purification of proteins from biological specimens.

## 4. Saccharide Affinity Electrophoresis

**Figure 3:**
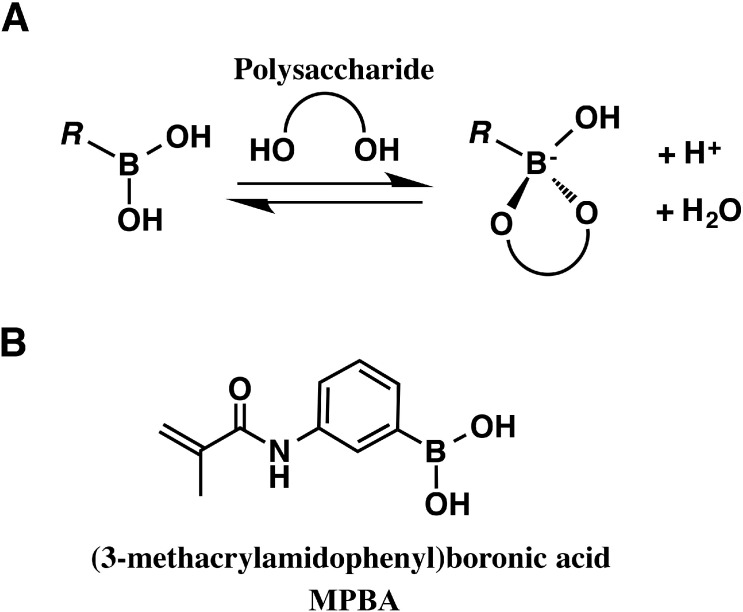
Saccharide affinity electrophoresis. (**A**) Reversible bonding between a boron compound and a polysaccharide; (**B**) the affinity probe MPBA.

Among the various posttranslational modifications of proteins, glycosylation is a widespread type of modification that plays an important role in protein biochemistry. The field of glycoprotein studies is called glycoproteomics and has recently gained importance. In developing their technique of boronate affinity saccharide electrophoresis, Jackson *et al.* utilized the affinity of saccharides for boron compounds [12]. Figure 3A shows the mechanism of reversible bonding of a saccharide to a boron compound. In boronate affinity saccharide electrophoresis, the boron compound that operates as the affinity probe for specific saccharides (fructose) or linear polyalcohols of saccharide derivatives containing sialic acids is immobilized by copolymerization in a polyacrylamide gel. The saccharides that have affinities for the boron compound migrate more slowly through this gel as a result of interactions with the boron compound. Figure 3B shows the molecular structure of the affinity probe [3-(methacryloylamino)phenyl]boronic acid (MPBA). By using an acrylamide-MPBA copolymer as a gel, Jackson *et al.* successfully separated low molecular weight saccharides, including monosaccharides and disaccharides, according to the differences in their structures [12]. Morais *et al.* applied this method to an analysis of glycoproteins and succeeded in separating glycoproteins in samples from diabetic patients [13]. Aikyo and Oh-Ishi developed a separation method in which differences in the sugar chains of glycoproteins are reflected in differences in their mobilities [14]. Because proteins have molecular weights of the order of tens of thousands to hundreds of thousands of Daltons and sugar chains have molecular weights of the order of several thousand Daltons, it is difficult to separate glycoproteins by SDS-PAGE solely on the basis of differences in the structures of their sugar chains. Aikyo and Oh-Ishi therefore attempted to analyze differences in sugar chain structures attached to a selected protein by means of two-dimensional (2D) gel electrophoresis (or diagonal electrophoresis) in which the first dimension was isoelectric focusing with an agarose gel [35] or SDS-PAGE and the second dimension was SDS-PAGE on an MPBA-immobilized gel. Because MPBA has different affinities for different saccharides, the separation and analysis of various glycoproteins is possible by this method.

## 5. Supported Molecular Matrix Electrophoresis and Its Application to Affinity Electrophoresis

**Figure 4:**
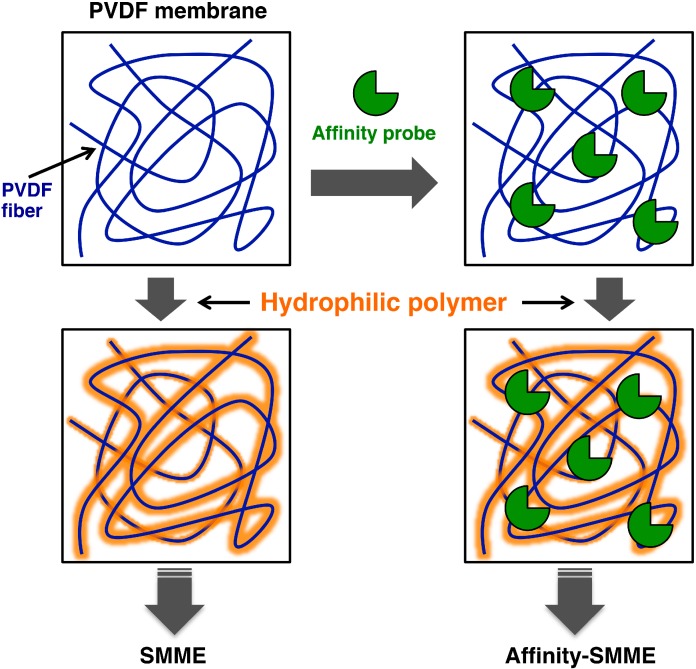
Schematic representation of supported molecular matrix electrophoresis (SMME). SMME uses a hydrophobic membrane (PVDF) that adsorbs proteins as a separation medium for electrophoresis after hydrophilization (left). By using this principle, a separation medium for affinity electrophoresis can be easily prepared by hydrophilizing the PVDF membrane after it has adsorbed biological components, such as proteins, that act as affinity probes (right).

Mucins are viscous glycoproteins produced by epithelial cells. It has been reported that changes in the structures of the sugar chains of mucins are related to the presence of tumors. However, because mucins are giant proteins with molecular weights of several megadaltons and sugar chains, which account for 50%–80% of this molecular weight, hinder the degradation of mucins by proteases, research on mucins has lagged behind recent developments in clinical proteomics, especially those in glycoproteomics and its associated analytical technologies. To permit the analysis of mucins, Matsuno *et al.* established a membrane electrophoresis technique that they named supported molecular matrix electrophoresis (SMME) [22,23,24]. In this method, fibers of a porous hydrophobic PVDF membrane, used as a supported matrix, are coated with hydrophilic poly (vinyl alcohol), which operates as a separation carrier (Figure 4). Although this method is similar to classical cellulose acetate electrophoresis, it differs in several respects, in that byproducts (for example, glucose fragments from cellulose) that might perturb sugar chain analysis are not produced during a glycosidase treatment, because a chemically-stable hydrophobic PVDF membrane is used, permitting proteins that remain on the membrane after migration to be detected by the use of antibodies or lectins. On the basis of band positions in SMME and subsequent mass spectrometric analysis, Matsuno *et al.* showed that the sugar chain profiles of mucins differ depending on the type of tumor tissue from which they are derived [22,23,24].

Matsuno and Kameyama have developed a technique that combines affinity electrophoresis with supported molecular matrix electrophoresis (affinity-SMME) and that utilizes the hydrophobic interactions between a PVDF membrane and biomolecules [25]. A separation carrier for affinity-SMME is prepared by allowing a PVDF membrane to adsorb substances, such as proteins or lipids, that are capable of hydrophobic interactions and which act as affinity probes and then coating the resultant membrane with a hydrophilic polymer (Figure 4). When the prepared membrane is used in electrophoresis, substances that interact with the molecules adsorbed on the membrane migrate more slowly. By using PVDF membranes onto which lectins, glycolipids or antibodies are adsorbed, Matsuno and Kameyama attempted to detect weak interactions between sugar chains and substances that recognize sugar chains. Because various affinity probes can be readily immobilized, this technique might provide breakthroughs in the search for glycoproteins capable of acting as new biomarkers for various diseases.

## 6. Phosphate Affinity Electrophoresis

**Figure 5:**
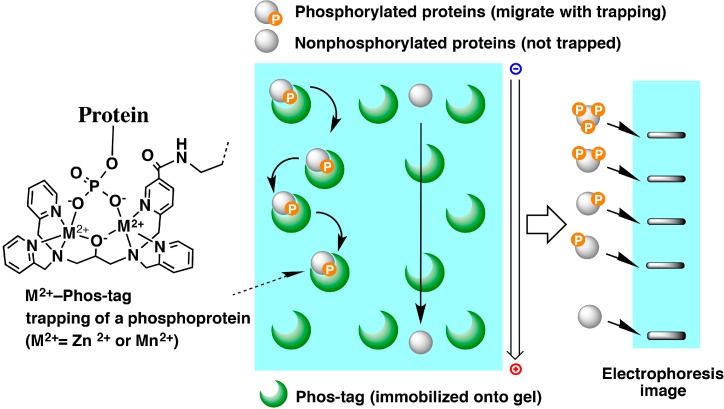
Schematic representation of phosphate affinity electrophoresis (Phos-tag SDS-PAGE). Copolymerization of acrylamide-pendent Phos-tag with acrylamide provides an SDS-PAGE gel that can specifically bind phosphorylated proteins migrating in the gel. This permits the separation of multiple phosphorylated forms of a protein, allowing quantitative analysis of the overall phosphorylation state and providing information on the differences in the functions of proteins arising from differences in their phosphorylation states.

We have been engaged in developing a technique known as phosphate affinity electrophoresis (Phos-tag SDS-PAGE), in which Phos-tag [36], a molecule that binds specifically to divalent phosphate ions in neutral aqueous solution, is used as an affinity probe. In this method, a gel in which the acrylamide-pendent Phos-tag monomer is copolymerized is used as a separation gel. Phosphorylated proteins migrate more slowly through the gel than do the corresponding non-phosphorylated proteins, because the phosphorylated proteins migrate with repeated reversible formation and breaking of bonds with the Phos-tag moieties immobilized on the gel (Figure 5). In this method, even similar protein molecules with identical numbers of phosphorylated amino acid residues, but which are phosphorylated at different amino acid sites, exhibit different mobility patterns and can be detected as separate bands [27,37]. In other words, this method can reveal differences in the phosphorylation states of a given protein. In addition, this method is capable of simultaneously visualizing various phosphorylated forms of a protein, including non-phosphorylated forms, and of quantifying their ratios.

By applying the principle of the Phos-tag system to the widely-used Laemmli SDS-PAGE system [38], we developed the Phos-tag SDS-PAGE system, which we first described in a publication of 2006 [11]. Because the Laemmli method involves the use of an alkaline electrophoresis gel with a pH of above nine, we used the Mn^2+^-Phos-tag complex (Figure 5, M^2+^ = Mn^2+^), which is capable of binding to phosphate groups under such conditions. Because this method is simple in operation and is compatible with the equipment and reagents used for conventional SDS-PAGE, it has become popular among researchers. This system has the additional advantage that, even if phospho-specific antibodies against proteins of interest cannot be obtained, phosphorylation states can be analyzed, provided antibodies against the non-phosphorylated proteins are available. This system has therefore been used for the analysis of the phosphorylation states of many proteins, and a number of valuable findings have been reported [39].

With regard to phosphorylated proteins *in vivo*, comprehensive identifications of phosphorylated proteins and determinations of phosphorylation sites have become practicable through the application of shotgun proteomics, because methods for concentrating and purifying phosphorylated peptides have been improved and techniques for mass spectrometry have become increasingly sophisticated. However, although shotgun proteomics can be used to perform comprehensive high-throughput analyses of phosphorylated proteins, the technique cannot accurately clarify differences in the phosphorylation states of individual proteins. Because it complements this disadvantage of shotgun proteomics, our Phos-tag SDS-PAGE system is expected to make a strong contribution to phosphoproteomics.

### 6.1. Examples of Analyses of Phosphorylated Proteins by Mn^2+^-Phos-Tag SDS-PAGE

Kimura *et al.* characterized the phosphorylation states of posttranslationally modified proteins by 2D electrophoresis with isoelectric focusing in the first dimension, Mn^2+^-Phos-tag SDS-PAGE in the second dimension and mass spectrometry after electrophoresis [40]. They examined the heterogeneous nuclear ribonucleoprotein K (hnRNP K), which is involved in events, such as chromatin remodeling, transcription and translation or mRNA processing, and they revealed that multiple forms of hnRNP K exist that have different phosphorylation states in terms of the numbers and the locations of the phosphate groups. Hosokawa *et al.* succeeded in measuring *in vivo* phosphorylation states of cyclin-dependent kinase 5 (Cdk5) activator p35 (Cdk5-p35), which regulates the cell cycle of proliferating cells [41]. Although Cdk5-p35, which has multiple phosphorylation sites, produced a complex banding pattern in Mn^2+^-Phos-tag SDS-PAGE, a combination of the technique with site-specific mutation of amino acids allowed the researchers to match the phosphorylation states to the banding patterns. In addition, they quantified the ratios of the individual *in vivo* phosphorylated forms.

Quantification of multiple phosphorylated forms of myosin light chain, which is difficult to perform by using conventional PAGE systems, can be readily performed by using the Mn^2+^-Phos-tag SDS-PAGE system, and many researchers worldwide have used this system to achieve quantitative analyses of the myosin light chain [42,43,44,45].

### 6.2. Development of an Improved Protocol, Neutral Phos-Tag SDS-PAGE

Since the early stages in the development of the Mn^2+^-Phos-tag SDS-PAGE system (see Section 6.1), we had known that some phosphorylated forms of proteins cannot be detected by using this system [37]. Because Phos-tag was originally designed to use a zinc complex that binds optimally to phosphate groups in neutral aqueous solutions, the conditions for the Mn^2+^-Phos-tag SDS-PAGE system were not optimal. We therefore used a Zn^2+^-Phos-tag complex (Figure 5, M^2+^ = Zn^2+^) in an SDS-PAGE system under neutral conditions to solve this problem. This improved system shows a significantly greater ability to separate many proteins, and it permits detailed analyses to be made of the phosphorylation states of proteins *in vivo*. Moreover, it facilitates the use of 2D difference gel electrophoresis (2D-DIGE) and is expected to advance detailed comprehensive analyses of phosphorylated forms of proteins in cells. We have named this improved protocol “neutral Phos-tag SDS-PAGE”. If you are interested in advances in our Phos-tag SDS-PAGE system, please refer to our recent papers on the subject [30,31,32]. The neutral Phos-tag SDS-PAGE is more durable, and it permits the development of a precast gel with an assured long-term quality and shelf life. Recently, the neutral Phos-tag SDS-PAGE precast gels have been commercialized by Wako Pure Chemical Industries, Ltd. (Osaka, Japan) as SuperSep Phos-tag [33]. Use of the precast gel is worthy of consideration in terms of labor saving, time saving and reliability in the detection of phosphoproteins.

### 6.3. Analysis of Chemically Unstable Histidine- or Aspartic Acid-Phosphorylated Proteins

Among the intracellular signaling systems of bacteria and plants are two-component signaling systems that are mediated by phosphorylations of histidine (His) and aspartic acid (Asp) residues. However, because of the chemical instability of phosphorylated His and Asp species, few methods are available for their analysis, and consequently, data on proteins containing such species are scarce compared with those for proteins phosphorylated on serine, threonine or tyrosine residues. By using our Mn^2+^- and Zn^2+^-Phos-tag SDS-PAGE systems, we demonstrated that quantitative analyses of the phosphorylation states of His and Asp residues are possible. We also succeeded in detailed measurements of time-dependent changes in transphosphorylation from His to Asp in a histidine kinase and a response regulator that serve as signaling molecules in bacteria [37,46,47]. More recently, many other research groups have also demonstrated the utility of the Phos-tag SDS-PAGE system for profiling of His- and Asp-phosphorylation in various two-component signaling systems [48,49,50,51,52,53].

In other attempts to solve the problem of the instability of phosphate groups on His and Asp, methods that rely on thiophosphorylation and subsequent monitoring of thiophosphorylated His and Asp residues rather than the corresponding phosphorylated residues have also been proposed [54,55,56,57]. By using ATPγS, the sulfur analogue of ATP, for phosphorylation *in vitro*, we examined the transphosphorylation of histidine kinase with a response regulator, and we attempted to analyze the reaction by using the Phos-tag SDS-PAGE system. Like phosphorylated residues, the corresponding thiophosphorylated residues were also found to bind to Phos-tag [47]. We then compared the functions of the phosphorylated and thiophosphorylated forms of the appropriate molecules. As a result, we found that the efficiency of transfer of thiophosphate groups from His to Asp was markedly low and that the use of thiophosphate groups instead of phosphate groups is not suitable for clarifying the original behavior of the molecules in some cases. However, the fact that phosphate groups, which should transfer promptly, were detected on His residues in the case of thiophosphate groups indicated that the reaction intermediates had been made visible. We believe that a combination of the Phos-tag SDS-PAGE system with thiophosphorylation might permit the detection of reaction intermediates of transphosphorylation between many enzymes and their substrates.

### 6.4. Development of Microchip-Based Phos-Tag Electrophoresis

Han *et al.* synthesized a linear copolymer of acrylamide-pendent Phos-tag and dimethylacrylamide as a separation carrier that they used to fill the channel of a microchip carrier by autonomous injection to form a microchip suitable for use in phosphate affinity electrophoresis [18,19,20]. This method is extremely useful for rapid quantitative analysis of the phosphorylation/dephosphorylation ratios of substrate peptides after *in vitro* kinase or phosphatase reactions. It has been considered for use as a microsystem for the determination of intracellular enzyme activities in the diagnosis of diseases, including cancers. Because microchip-based electrophoresis is suitable for high-speed separation of minute amounts of samples and because data visualization and data analysis are integrated in the chip itself, microchip-based electrophoresis is expected to become widely used as an alternative method to complicated slab-gel electrophoresis. The principle of Phos-tag-based phosphate affinity electrophoresis will be applied not only to microchip-based electrophoresis, but also to other electrophoresis systems, and this should lead to advancements in easy and simple systems for the analysis of phosphorylation.

## 7. Conclusions and Perspectives

As a result of the recent remarkable development of technologies in proteomics, it is now possible to obtain large quantities of accurate data quickly. However, there are many biomolecules, including the posttranslationally modified proteins that play important roles *in vivo*, that have not yet been studied because of difficulties in their analyses. Affinity electrophoresis techniques have made major breakthroughs in studies involving the detailed analyses of such biomolecules. One of the advantages of using affinity electrophoresis is that the separated protein samples can be fixed in an electrophoresis matrix, such as a polyacrylamide gel or PVDF membrane, and then, the target molecules can be visualized selectively by various staining and multiple immunoblotting techniques. Furthermore, affinity electrophoresis techniques have made important contributions to the analyses of reversible intermolecular interactions that play key roles in a wide variety of biological systems. Because the scope of affinity probes is limitless, ingenious combinations of affinity probes with electrophoresis techniques should eventually reveal the behaviors of a range of biomolecules.
